# Digital technology applications for contact tracing: the new promise for COVID-19 and beyond?

**DOI:** 10.1186/s41256-020-00164-1

**Published:** 2020-08-03

**Authors:** Priscilla N. Owusu

**Affiliations:** Heidelberg University, Institute of Global Health, Heidelberg, Germany

**Keywords:** COVID-19, Digital health technology, Population health, Program implementation, Health policy

## Abstract

Among the most critical strategies in the fight against the Corona Virus Disease (COVID-19) is the rapid tracing and notification of potentially infected persons. Several nations have implemented mobile software applications (“apps”) to alert persons exposed to the coronavirus. The expected advantages of this new technology over the traditional method of contact tracing include speed, specificity, and mass reach. Beyond its use for mitigating and containing COVID-19, digital technology can complement or even augment the traditional approach to global health program implementation. However, as with any new system, strong regulatory frameworks are necessary to ensure that individual information is not used for surveillance purposes, and user privacy will be maintained. Having safeguarded this, perhaps the global health community will witness the beginning of a new era of implementing mass health programs through the medium of digital technology.

## Background

On 30 January 2020, the World Health Organization (WHO) [1] declared the novel pneumonia-like disease caused by the severe acute respiratory syndrome coronavirus-2 **(**SARS-CoV-2**)** a Public Health Emergency of International Concern (PHEIC). Approximately 6 weeks later, the WHO determines this outbreak to be a global pandemic. Per official reports on 8 May 2020, the new coronavirus disease (officially termed COVID-19) has been responsible for 3,866,642 total confirmed cases and 270,118 global deaths [2]. As the world battles the COVID-19 pandemic, contact tracing has been critically important among the repertoire of solutions to help “flatten the curve” of coronavirus infections. This commentary discusses the emerging use of digital health technology for the purpose of COVID-19 contact tracing, the latent benefits of adopting this model into other areas of global health, and the urgency of robust local and international data protection regulatory frameworks.

## Conventional method of contact tracing

Contact tracing is a monitoring process for individuals who have been exposed to someone infected with a virus, and are at higher risk of infecting themselves and others [3]. The process involves three basic steps, namely **contact identification,** where the infected person recalls activities and the roles of persons involved since the onset of the infectious disease; **contact listing**, which provides the names of potentially infected contacts, and **contact follow-up**, to monitor any onset of symptoms associated with the viral infection [3].

Contact tracing has conventionally been implemented by an “Investigation Team” through an iterative process of interviews with the case, or if unable to talk or death has occurred, then with his or her family members [4]. The team proceeds to locate and notify all persons identified as contacts with the infected person. This investigative method of contact tracing may be inherently challenging in that it poses a logistical burden on the Investigation Team, is time consuming, and potentially stigmatizes the contacts and their associations who may wish to maintain the private status of their infection.

## Emerging use of digital technology for COVID-19 contact tracing

The phenomenon of commissioning mobile software technology for contact tracing is not unusual. In 2010, a consortium of researchers in the United Kingdom allowed users to install a mobile software application called the *FluPhone* [5] to anonymously collect information on social encounters using Bluetooth, GPS coordination, and self-reported data. Successively, this information aided in developing predictive models of the influenza virus transmission patterns within each community.

Amid the COVID-19 outbreak, as many as 25 countries [6] (Table 1) across the globe have embraced digital technology as an aggressive measure to combat the spread of the coronavirus.

**Table 1:** Worldwide implementation of digital health technology for COVID-19 contact tracing

Country	Description	Technology Feature	Reference
Austria	*Stopp Corona* app. Available by voluntary download for Apple iOS and Android software devices.	Bluetooth, and API to notify users of potential exposure.	Stopp Corona: Austrian Red Cross. https://participate.roteskreuz.at/stopp-corona/ (2020) Accessed 22 June 2020.
Australia	*COVIDSafe* app. Available on Apple iOS and Android devices.	Bluetooth technology that notes the date, time, distance and duration of contact with other users. Users are notified of potential exposure.	Australian Government, Department of Health: COVIDSafe App. https://www.health.gov.au/(2020). Accessed 22 June 2020.
China	Close contact detector app. Provides users with unique QR codes.	Interfaces with other widely-used apps such as *WeChat*, *Alipay* and *QQ*.	XinhuaNet: China introduces novel coronavirus close contact detection app. http://www.xinhuanet.com/english/2020-02/10/c_138770415.htm (2020). Accessed 8 May 2020.
Germany	*Corona-Warn* app. Voluntary download; available on Apple iOS and Android software devices.	Bluetooth API technology to scan identification codes on nearby phones. Notification to user upon exposure to proximal code.	Corona-Warn-App Open Source Project. https://www.coronawarn.app/en/ (2020) Accessed 22 June 2020.
Ghana	*GH Covid-19 Tracker* app. Available by voluntary download for Apple iOS and Android software devices.	Bluetooth and GPS technology, geographically enabled features, which provides detailed information on event, location after potential exposure.	Ministry of Communications, Ghana: Launch of GH COVID-19 tracker app.https://www.moc.gov.gh/launch-gh-covid-19-tracker-app(2020) Accesed 26 April 2020.
Singapore	*TraceTogether* app. Widely received among citizenry (70% support).	Operates under Bluetooth technology.	Government of Singapore:TraceTogether Singapore. https://www.tracetogether.gov.sg/ (2020). Accessed 26 April 2020.
Switzerland	*SwissCovid* app. Available from 25 June 2020 for Apple iOS and Android software devices. Voluntary download.	Bluetooth technology recording previous users in close contact. Cantonal authorities notify other users of exposure.	Federal Office of Public Health (FOPH): New coronavirus: SwissCovid app and contact tracing. https://www.bag.admin.ch/bag/en/ (2020). Accessed 22 June 2020.

The common approach to this new infrastructure involves the usage of Bluetooth and application programming interfaces (APIs) provided by Google and Apple to enable interaction between mobile devices in close proximity. Bluetooth communication features in iOS and Android devices will assign a unique, anonymous identification code for all contacts in close proximity of a person’s device. Upon consent, if one tests positive for COVID-19, the app downloads a history of these identification codes for public health authorities to notify the close contacts in an effort to break the chain of transmission.

## Extensive benefits of digital infrastructure application for global health

For some global health experts, the use of digital technology in the context of COVID-19 marks the ushering in of a promising new milestone in the implementation of mass interventions. Besides contact tracing, diverse digital infrastructure taking the form of Internet of Healthcare Things (IoHT), big data, and machine learning have played intergral roles in the efficient prevention and management of the new SARS-CoV-2 disease [7, 8]. Present applications of this technology are expanding to include the development of precision treatments for patients with COVID-19, streamlining of clinical workload, drug and vaccine discovery efforts, and predictive analytics to forecast the trajectory of outbreaks [7, 8].

Broadly considered, digital and AI-based interventions for global health replicate the basic principles of health programming in key ways. Fundamentally, it respects individual autonomy through opt-in/opt-out features which allow target populations to indicate or refuse consent to participate. Secondly, digital technology minimizes the burden of participation by eliminating the need for continuous self-reporting. Thirdly, automated processes circumvent any recall bias from the infected person, in addition to other potential human errors and gaps in data reporting. Lastly, and perhaps most important for the infected persons is the advantage of reducing the stigmatizing effect of face-to-face interviews with the official contact tracing Investigation Team.

## Recommendations on regulatory frameworks for data privacy and protection

Despite the promise of digital interventions for global health, precautionary measures must be responsibly exercised. Governing authorities are to ensure that policy frameworks include strong protections for the privacy of users. By the same token, international or national laws should guarantee that data collected in the interest of public health are not ulteriorly used for retaliatory or surveillance purposes.

Established norms and principles governing digital technology and telecommunications also matter in the “context of international security” [8]. With growing internet use, criminal activity by hackers have frequently targeted vital State and civilian information in the cyberspace. Moreso in extreme cases, such as international armed conflicts, direct attacks on digital infrastructure enabling the delivery of public services can cause deliberate harm. Thus international humanitarian laws ought to be amended to govern responsible State behavior concerning civilian information available in the cyberspace. International laws should oblige States to enact protective measures to prevent cyberattacks on digital infrastructure [9].

Of critical note, it is recommended that at the design stage digital program implementation specialists carefully consider any potential threats against the proper inclusion of vulnerable populations. Preventive measures against the exploitation of these populations may incorporate the simplification of consent language or the availability of audio/visual translations for handicapped persons.

## Conclusion

Digital technology together with AI and mass data have arrived in full force at the doorsteps of global health in the ongoing fight against the COVID-19 pandemic. With support from national governments, these technology applications serve as an alert system for enabling rapid contact tracing and notification as well as mass reach to the population. For epidemiologists and behavioral scientists, the mass data harvested from these digital platforms present an immense repository of evidence that can be beneficial in informing preparative steps for future pandemics. Beyond its use for mitigating and containing COVID-19, digital technology can complement or in some cases amplify the traditional approach to global health program implementation. However, as with any new system, extra measures such as privacy protection, and skills training for specialists, are warranted to avoid blindspots which could overshadow the benefits.

## Data Availability

Not applicable.

## Abbreviations

COVID-19: Corona virus disease 2019
APIs: Application programming interfaces
AI: Artificial intelligence
